# Multiparametric approach with synthetic MR imaging for diagnosing salivary gland lesions

**DOI:** 10.1007/s11604-024-01578-4

**Published:** 2024-05-11

**Authors:** Koji Takumi, Ryota Nakanosono, Hiroaki Nagano, Hiroto Hakamada, Fumiko Kanzaki, Kiyohisa Kamimura, Masatoyo Nakajo, Yukari Eizuru, Hiromi Nagano, Takashi Yoshiura

**Affiliations:** 1 Department of Radiology, Kagoshima University Graduate School of Medical and Dental Sciences, 8-35-1 Sakuragaoka, Kagoshima City, 890-8544 Japan; 2 Department of Otolaryngology Head and Neck Surgery, Kagoshima University Graduate School of Medical and Dental Sciences, 8-35-1 Sakuragaoka, Kagoshima City, 890-8544 Japan

**Keywords:** Salivary gland tumors, Synthetic MRI, T1 map, T2 map, Proton density

## Abstract

**Purpose:**

To determine whether synthetic MR imaging can distinguish between benign and malignant salivary gland lesions.

**Methods:**

The study population included 44 patients with 33 benign and 11 malignant salivary gland lesions. All MR imaging was obtained using a 3 Tesla system. The QRAPMASTER pulse sequence was used to acquire images with four TI values and two TE values, from which quantitative images of T1 and T2 relaxation times and proton density (PD) were generated. The Mann–Whitney *U* test was used to compare T1, T2, PD, and ADC values among the subtypes of salivary gland lesions. ROC analysis was used to evaluate diagnostic capability between malignant tumors (MTs) and either pleomorphic adenomas (PAs) or Warthin tumors (WTs). We further calculated diagnostic accuracy for distinguishing malignant from benign lesions when combining these parameters.

**Results:**

PAs demonstrated significantly higher T1, T2, PD, and ADC values than WTs (all *p* < 0.001). Compared to MTs, PAs had significantly higher T1, T2, and ADC values (all *p* < 0.001), whereas WTs had significantly lower T1, T2, and PD values (*p* < 0.001, *p* = 0.008, and *p* = 0.003, respectively). T2 and ADC were most effective in differentiating between MTs and PAs (AUC = 0.928 and 0.939, respectively), and T1 and PD values for differentiating between MTs and WTs (AUC = 0.915 and 0.833, respectively). Combining T1 with T2 or ADC achieved accuracy of 86.4% in distinguishing between malignant and benign tumors. Similarly, combining PD with T2 or ADC reached accuracy of 86.4% for differentiating between malignant and benign tumors.

**Conclusions:**

Utilizing a combination of synthetic MRI parameters may assist in differentiating malignant from benign salivary gland lesions.

**Supplementary Information:**

The online version contains supplementary material available at 10.1007/s11604-024-01578-4.

## Introduction

Salivary gland tumors present a unique diagnostic challenge due to their diverse histopathological characteristics. Most of these lesions are benign, including pleomorphic adenomas (PAs) and Warthin’s tumors (WTs), and each have distinct clinical behaviors and treatment approaches [1]. WTs are usually treated with enucleation or conservative management because of their low potential for malignancy compared to PAs and malignant tumors (MTs) [2]. For PAs, radical surgical excision to reduce the rate of recurrence is the recommended treatment approach [3]. In addition, the risk of malignant transformation in PAs is 1.5% during the initial five years and PAs have recurrence rates ranging from 6.7 to 45% [4]. In contrast, total excision with radiotherapy is usually applied for MTs, after which the facial nerve may be lost [5]. The treatment of malignant salivary gland lesions requires a diverse approach according to the tumor's histopathological characteristics and the patient's risk classification [5]. For instance, high-grade tumors or those exhibiting aggressive behavior, such as adenoid cystic carcinomas and high-grade mucoepidermoid carcinomas, necessitate more extensive surgical margins with neck dissection (or irradiation). This is usually complemented by adjuvant radiotherapy to reduce the risk of local recurrence. Additionally, based on the extent of disease progression, systemic chemotherapy, including immunotherapy, may also be selected. Conversely, low-grade tumors such as basal cell adenocarcinomas and secretory carcinomas might be managed effectively with surgery alone, given their slower growth rate and lower metastatic potential. On the other hand, the treatment of salivary gland lymphomas depends on histology and staging of the disease. Treatment modalities for salivary gland lymphomas include surgery, radiation therapy, and chemotherapy, either alone or as a multimodality treatment [6]. Accordingly, the distinction between benign and malignant lesions and the accurate diagnosis of various subtypes is crucial for appropriate treatment planning.

Conventional MRI that employs T1-weighted and T2-weighted images is commonly used to evaluate and differentiate between various types of salivary gland lesions [7–10]. Typically, benign tumors (BTs) appear as well-circumscribed, smooth masses. In contrast, MTs tend to have irregular or ill-defined margins with a tendency to infiltrate adjacent structures, and they often display heterogeneous signal patterns on both T1-weighted and T2-weighted images due to the presence of necrotic or hemorrhagic areas [8, 11]. Both dynamic contrast-enhanced MR imaging and apparent diffusion coefficient (ADC) values derived from diffusion-weighted imaging (DWI) have also been reported as useful tools in diagnosing salivary gland lesions [12–15]. Rapid contrast enhancement with a low washout ratio has been reported as highly suggestive of malignancy [13], whereas WTs show rapid contrast enhancement with a high washout ratio and PAs show gradual, persistent enhancement [13]. ADC values of PAs on diffusion-weighted imaging (DWI) have been reported to be significantly higher than those of WTs or MTs [12, 15]. However, in both of these methods there are considerable overlaps between benign and malignant lesions, and between subtypes of salivary gland lesions [12, 14, 15].

Synthetic MRI is an innovative technique now introduced into clinical practice that acquires multiple parameters in a single scan, generating T1 and T2 relaxation times as well as proton density (PD) maps [16]. The advantage of this technology lies in its ability to provide high-precision imaging data in a short time. Recent studies have reported preliminary findings regarding the utility of quantitative relaxation parameters derived from synthetic MRI in various organs, including the brain [16], breast [17], prostate [18], and rectum [19]. There have been no reports of utilizing synthetic MRI for the diagnosis of salivary gland tumors. This technique enables detailed capture of changes in tissue microstructure and water content that can aid in differentiating the subtypes and the malignancy of salivary gland lesions. Moreover, combining these parameters may lead to a deeper understanding of the histological and pathological characteristics. We hypothesized that parameters derived from synthetic MRI and their combinations could be useful for differentiating salivary gland lesions. Therefore, the purpose of this study was to determine whether the quantitative parameters derived from synthetic MRI and their combination can assist in the diagnosis of salivary gland lesions.

## Materials and methods

### Patients

This retrospective study was approved by our institutional ethics review board, and the requirement for patients’ informed consent was waived. A retrospective review of the MR imaging database and clinical records of our radiology department identified 108 consecutive patients who had undergone pretreatment MR examination of the salivary glands between March 2021 and September 2023. Among these, 47 patients met the following inclusion criteria: (1) synthetic MR images had been obtained, and (2) a pathological diagnosis had been obtained by biopsy or surgical resection. Two patients with predominantly cystic lesions were excluded. Finally, 44 patients (26 men and 18 women; mean age, 62.5 years; age range, 22–88 years) with a total of 44 salivary gland lesions (41 parotid and 3 submandibular gland lesions) were included in this study. Among these cases, 17 had undergone a biopsy prior to MRI imaging. The final pathological confirmation of diagnoses was achieved based on surgical specimens for 38 patients, while 6 patients were diagnosed via biopsy. Of the 44 lesions, 33 were BTs (12 PAs, 15 WTs, 3 basal cell adenomas, and 3 schwannomas) and 11 were malignant (3 malignant lymphomas, 2 carcinoma ex pleomorphic adenomas, one adenoid cystic carcinoma, one mucoepidermoid carcinoma, one squamous cell carcinoma, one secretory carcinoma, one basal cell adenocarcinoma, and one capicua transcriptional repressor (CIC)-rearranged sarcoma).

### MR imaging protocols and image processing

All MR examinations were conducted using a 3 Tesla system (Ingenia 3.0 T; Philips Healthcare, Best, The Netherlands) with a 20-channel standard phased-array head and neck coil. To minimize motion artifacts, careful attention was given to ensure patients’ comfort while positioned in the scanner, and they were instructed on the importance of remaining still during the scan. Additionally, the head was securely fixed within the coil to prevent movement during the scan.

For the acquisition of synthetic MRI data to measure relaxation times and PD, we used a two-dimensional axial method with QRAPMASTER (quantification of relaxation times and proton density by multi-echo acquisition of saturation-recovery using turbo spin-echo readout) pulse sequence. Two echo times (TE, 13 ms and 100 ms) along with four delay times (110/440/1210/2530 ms) were used to generate eight actual and eight imaginary images. Other parameters were as follows: repetition time (TR), 6100 ms; flip angle, 90°; sensitivity encoding factor, 3; field of view (FOV), 230 × 192 mm; matrix resolution, 512 × 512; echo-train length, 10; slice thickness and gap, 4 mm and 0.8 mm respectively; 20 slices; and scan time, 5 min and 54 s. Quantification map acquisition was performed with SyMRI software (Version 19.3; SyMRI, Linköping, Sweden).

DWI was also performed in the axial plane with a single-shot spin-echo echo planar imaging sequence with the following parameters: TR, 5000 ms; TE, 85 ms; FOV, 230 × 230 mm; matrix resolution, 120 × 120; slice thickness, 4 mm; *b* values = 0 and 800 (s/mm^2^); diffusion gradient direction, 3; scan time, 2 min and 25 s. The ADC value was calculated in a voxel-by-voxel manner by mono-exponential fitting with the pair of b-values.

### Quantitative analyses

T1, T2, PD, and ADC maps were measured by two radiologists with 22 and 5 years of radiology experience who were blinded to the final pathological results. With reference to the T1- and T2-weighted images, the mean values of T1, T2, PD, and ADC in each lesion were measured within a freehand region of interest (ROI) on the cross-sectional area of the largest lesion. To avoid the influence of the partial volume effect, the ROI was set slightly inside the margins of the lesion (Supplementary Fig.). Care was taken to avoid cystic or necrotic areas within lesions. All synthetic parameters were measured using the software (SyMRI version 19.3, SyntheticMR, Linköping, Sweden). ADC was measured using a medical imaging system (SYNAPSE SAI viewer version 2.2, Fujifilm Medical, Tokyo, Japan). The mean value of the two radiologists was obtained as the representative value of each lesion for all quantitative variables.

### Statistical analysis

Interobserver agreement for T1, T2, PD, and ADC measurement was evaluated using intraclass correlation coefficient (ICC) and Bland–Altman plot. ICCs were considered to indicate excellent agreement when > 0.74.

All continuous variables used in the analysis, except for T1 and T2 values of PAs, were normally distributed according to the Shapiro–Wilk test, however, T1 and T2 values of PAs did not follow a normal distribution. Therefore, T1, T2, PD, and ADC values of BTs and MTs were compared using the Mann–Whitney *U* test. These parameters were also compared among each subgroup using the Mann–Whitney *U* test. ROC curve analysis was performed to evaluate the ability of each parameter to differentiate MTs from PAs or WTs. For each parameter, optimal cutoff values were chosen using a threshold criterion that maximized the Youden index. The sensitivity, specificity, positive predictive value, negative predictive value, and accuracy in differentiating MTs from BTs were determined by combining the two most indicative parameters, as suggested by the area under the ROC curve (AUC) for differentiating MTs from PAs or WTs.

All data for continuous variables are presented as the mean ± standard deviation (SD). Values of *p* < 0.05 with Bonferroni correction were considered indicative of significant difference. Statistical analyses were performed using MedCalc version 19.4 (MedCalc Software, Mariakerke, Belgium) and SPSS version 25.0 (SPSS, Chicago, IL).

## Results

Interobserver agreement was excellent for all quantitative measurements (Table 1). The Bland–Altman analyses showed relatively small bias and 95% limits of agreement for each quantitative parameter (Table 1).

**Table 1:** Interobserver agreement

Quantitative parameters	ICC	Bland–Altman analysis (%)		
			95% limits of agreement	
		Bias	Lower	Upper
T1 (ms)	0.99 (0.98, 0.99)	– 51.22	– 271.80	169.36
T2 (ms)	0.93 (0.87, 0.96)	3.64	– 23.11	30.41
PD (%)	0.90 (0.82, 0.94)	1.53	– 5.73	8.79
ADC (× 10^−3^ mm^2^/s)	0.98 (0.96, 0.99)	0.00	– 0.19	0.20

*ICC* intraclass correlation coefficient, *PD* proton density, *ADC* apparent diffusion coefficient

Numbers in parentheses are 95% confidence intervals

### Synthetic MRI parameters and ADC values for salivary gland lesions

T1, T2, PD, and ADC values for PAs, WTs, and MTs were 2270 ± 670 ms, 118 ± 45 ms, 87 ± 7%, and 1.55 ± 0.36 × 10^−3^ mm^2^/s; 1079 ± 147 ms, 62 ± 4 ms, 74 ± 4%, and 0.85 ± 0.14 × 10^−3^ mm^2^/s; and 1512 ± 378 ms, 73 ± 12 ms, 83 ± 7%, and 0.93 ± 0.22 × 10^−3^ mm^2^/s, respectively. T1, T2, PD, and ADC values for all BTs were 1708 ± 754 ms, 94 ± 44 ms, 81 ± 8%, and 1.26 ± 0.50 × 10^−3^ mm^2^/s, respectively (Table 2).

**Table 2:** Comparison of MR parameters between benign and malignant salivary gland lesions

Parameters	MTs (*n* = 11)	All BTs (*n* = 33)	*p*^a^ (MTs vs. all BTs)	PAs (*n* = 12)	*p*^a^ (MTs vs. PAs)	WTs (*n* = 15)	*p*^a^ (MTs vs. WTs)
T1 relaxation time (ms)	1512 ± 378	1708 ± 754	0.936	2270 ± 670	** < 0.001**	1079 ± 147	** < 0.001**
T2 relaxation time (ms)	73 ± 12	94 ± 44	0.376	118 ± 45	** < 0.001**	62 ± 4	**0.008**
Proton density (%)	83 ± 7	81 ± 8	0.831	87 ± 7	0.079	74 ± 4	**0.003**
ADC (× 10^−3^ mm^2^/s)	0.93 ± 0.22	1.26 ± 0.50	0.057	1.55 ± 0.36	** < 0.001**	0.85 ± 0.14	0.540

*ADC* apparent diffusion coefficient, *MTs* malignant tumors, *BTs* benign tumors, *PAs* pleomorphic adenomas, *WTs* Warthin tumors

^a^Comparisons with malignant lesions were assessed using the Mann–Whitney *U* test

### Comparison of parameters among subtypes of salivary gland lesions

**Fig. 1:**
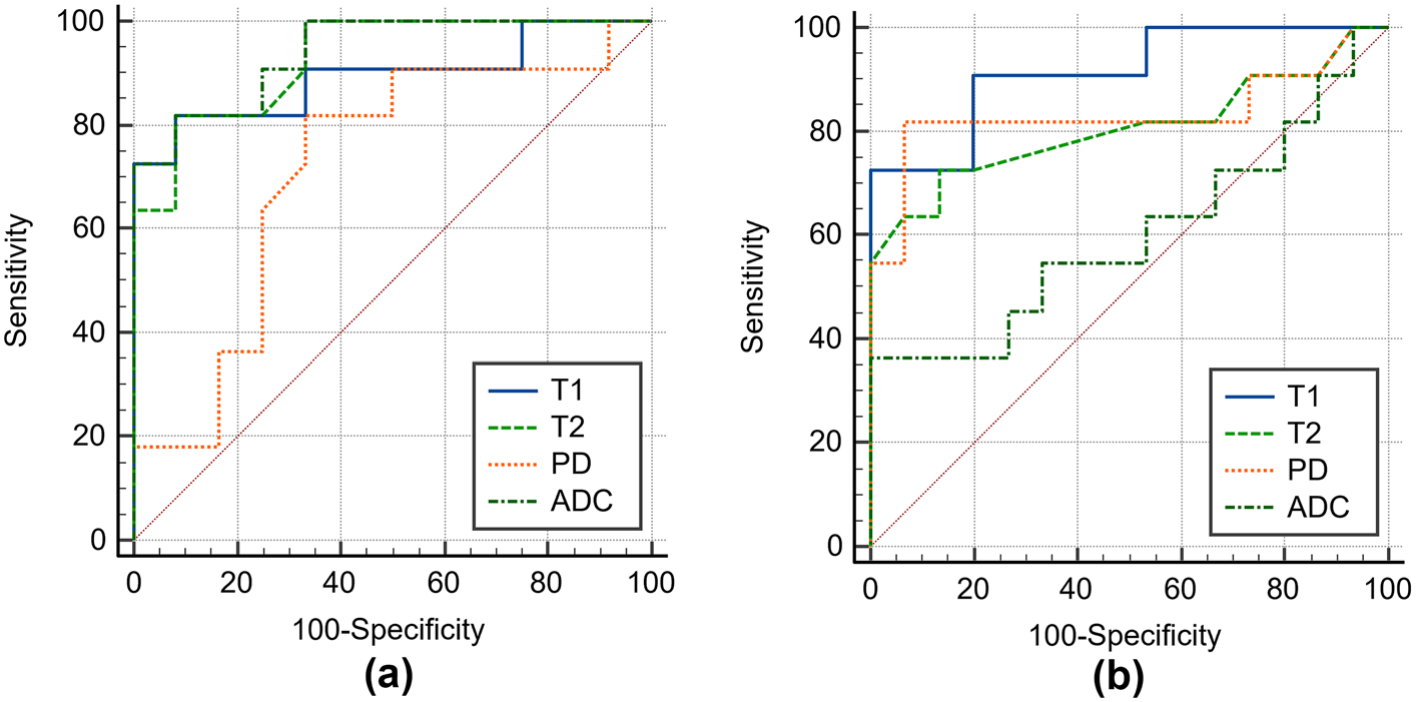
Receiver-operating characteristic (ROC) curves of T1, T2, PD, and ADC values for differentiating malignant tumors (MTs) from pleomorphic adenomas (PAs) (**a**) and Warthin tumors (WTs) (**b**). In differentiating MTs from PAs, area under the ROC curve (AUC) values for T1, T2, PD, and ADC were 0.894, 0.928, 0.716, and 0.939, respectively. In differentiating MTs from WTs, AUCs for T1, T2, PD, and ADC were 0.915, 0.806, 0.833, and 0.600, respectively

**Fig. 2:**
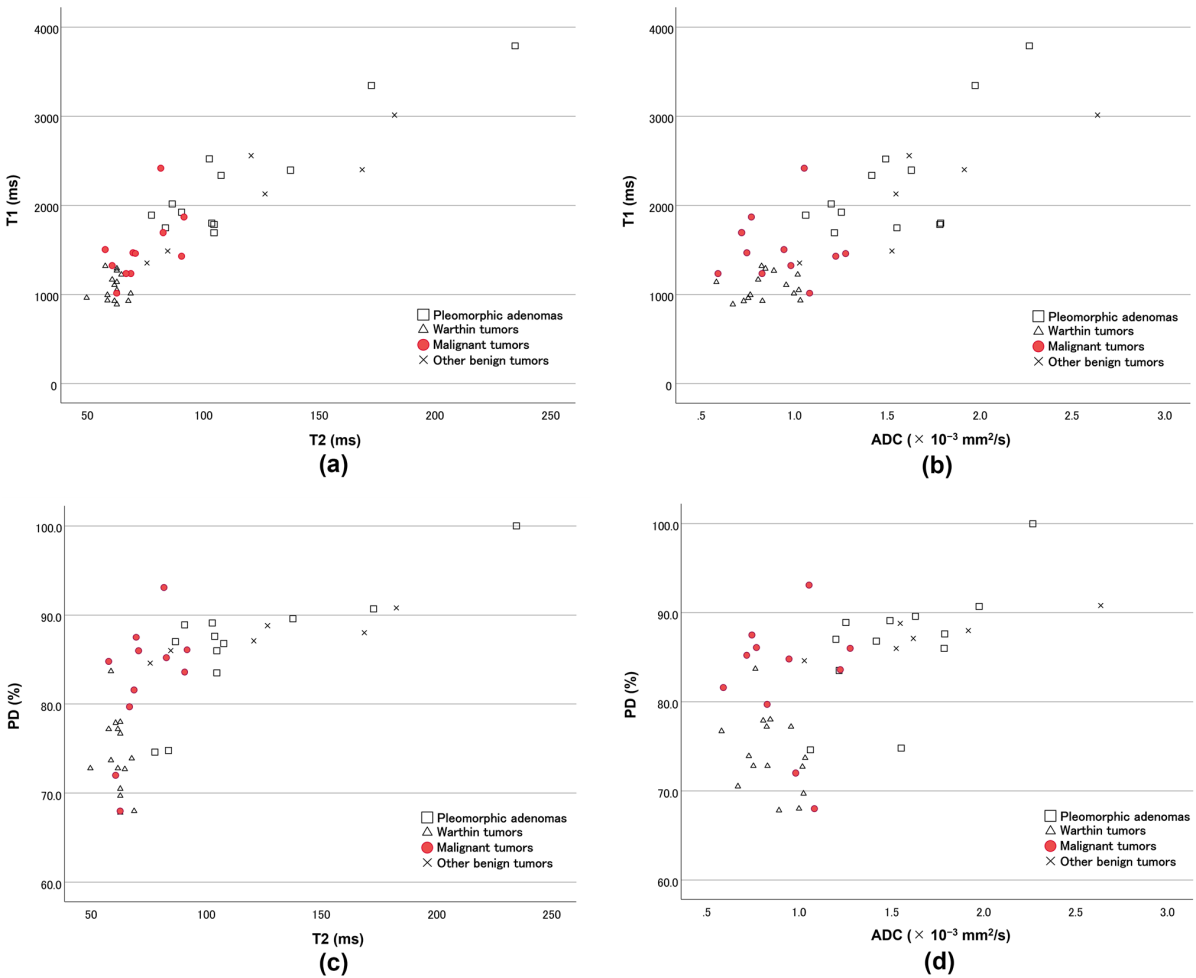
Scatterplots of T1 versus T2 (**a**), T1 versus ADC (**b**), proton density versus T2 (**c**), and proton density versus ADC (**d**) of various salivary gland lesions. Values of pleomorphic adenoma tend to cluster in the areas of high T1, T2, and ADC. Values of Warthin tumors tend to cluster in the areas of low T1, T2, and ADC. Values of malignant lesions tend to cluster in areas of low T2 and ADC compared to those of pleomorphic adenomas, and of high T1 compared to those of Warthin tumors

No significant difference was observed between BTs and MTs for any parameter (all *p* > 0.05). PAs demonstrated significantly higher T1, T2, PD, and ADC values than WTs (all *p* < 0.001). To differentiate PAs from WAs, AUC values for T1, T2, PD, and ADC were 1.000 with 95% CI of 0.872–1.000, 1.000 with 95% CI of 0.872–1.000, 0.928 with 95% CI of 0.760–0.991, and 1.000 with 95% CI of 0.872–1.000, and the optimal threshold values were ≤ 1317 ms, ≤ 69 ms, ≤ 78%, and ≤ 1.033 × 10^−3^ mm^2^/s, respectively. Compared to MTs, PAs had significantly higher T1, T2, and ADC values (all *p* < 0.001), whereas WTs had significantly lower T1, T2, and PD values (*p* < 0.001, *p* = 0.008, and *p* = 0.003, respectively) (Table 2). T2 and ADC values performed the best in differentiating between MTs and PAs (AUC = 0.928 with 95% CI of 0.740–0.994 and 0.939 with 95% CI of 0.756–0.996, respectively). T1 and PD values performed the best in differentiating between MTs and WTs (AUC = 0.915 with 95% CI of 0.738–0.988 and 0.833 with 95% CI of 0.638–0.949, respectively) (Fig. 1). The T1, T2, PD, and ADC values for each tumor type are plotted in Fig. 2. PAs typically had high T1, T2, PD, and ADC values; in contrast, WTs had low T1, T2, PD, and ADC values. Most MTs had lower T2 and ADC values compared to PAs, and higher T1 and PD values compared to WTs.

### Combination of parameters for differentiating between MTs and BTs

The combination of T1 (> 1317 ms) with T2 (≤ 83 ms) or ADC (≤ 1.08 × 10^–3^ mm^2^/s) achieved accuracy of 86.4% in distinguishing between MTs and BTs. Similarly, the combination of PD (> 78%) with T2 (≤ 83 ms) or ADC (≤ 1.08 × 10^–3^ mm^2^/s) reached accuracy of 86.4% in differentiating between MTs and BTs (Table 3).

**Table 3:** Performance of combinations of MR parameters in diagnosing malignant salivary gland lesions

Parameters	Threshold criterion	Sensitivity (%)	Specificity (%)	PPV (%)	NPV (%)	Accuracy (%)
T1 + T2	T1 > 1317 ms, T2 ≤ 83 ms	63.6	93.9	77.8	88.6	86.4
T1 + ADC	T1 > 1317 ms, ADC ≤ 1.08 × 10^–3^ mm^2^/s	63.6	93.9	77.8	88.6	86.4
PD + T2	PD > 78%, T2 ≤ 83 ms	63.6	93.9	77.8	88.6	86.4
PD + ADC	PD > 78%, ADC ≤ 1.08 × 10^–3^ mm^2^/s	63.6	93.9	77.8	88.6	86.4

*PD* proton density, *ADC* apparent diffusion coefficient, *PPV* positive predictive value, *NPV* negative predictive value

**Fig. 3:**
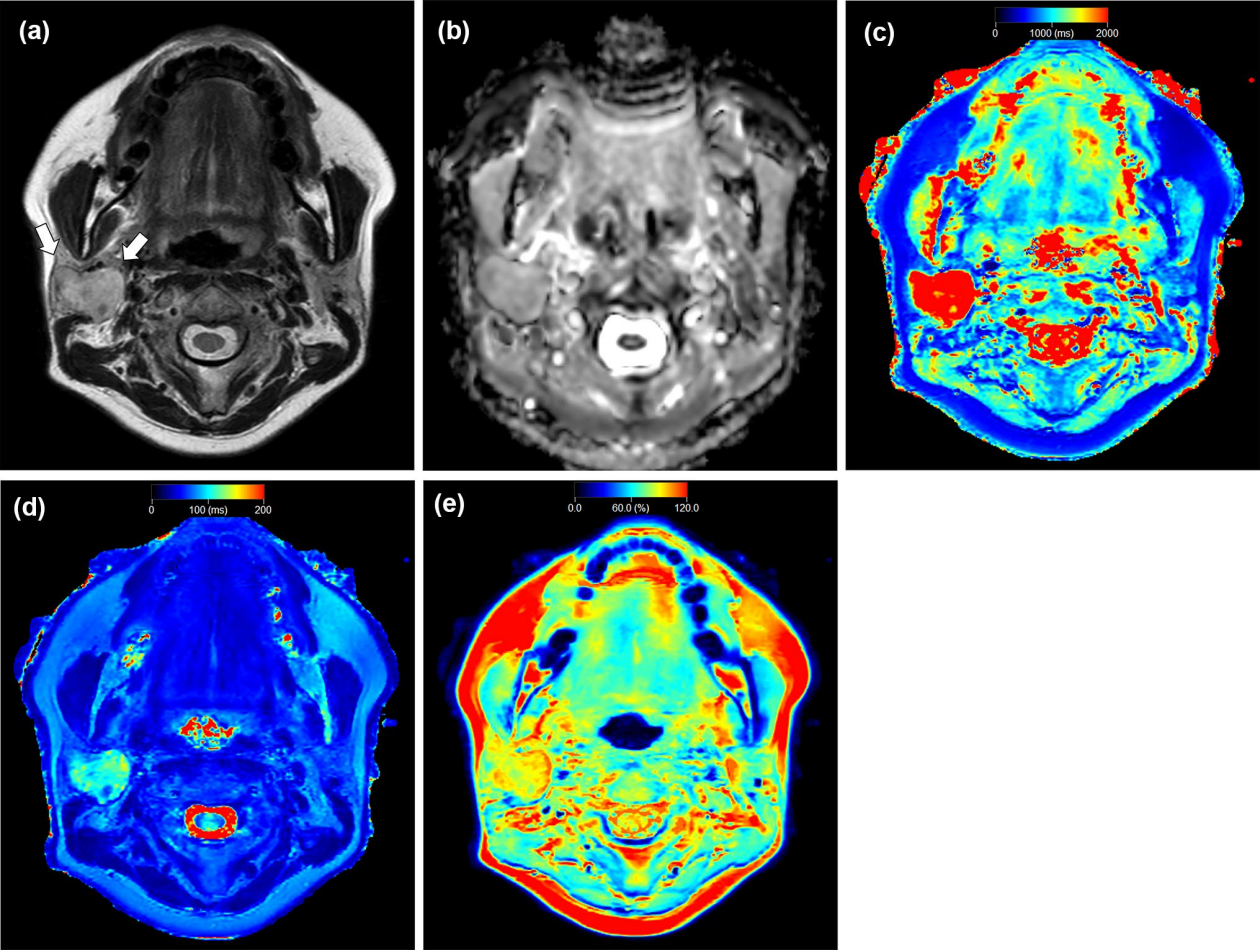
A 49-year-old female with pleomorphic adenoma. T2-weighted image (**a**) shows a lesion with higher signal intensity (arrow) relative to parenchyma in the adjacent right parotid gland. Apparent diffusion coefficient (ADC) (**b**), T1 (**c**), T2 (**d**), and PD (**e**) maps show the lesion, which has a mean ADC value of 1.49 × 10^−3^ mm^2^/s, mean T1 value of 2520 ms, mean T2 value of 103 ms, and mean PD value of 89%

**Fig. 4:**
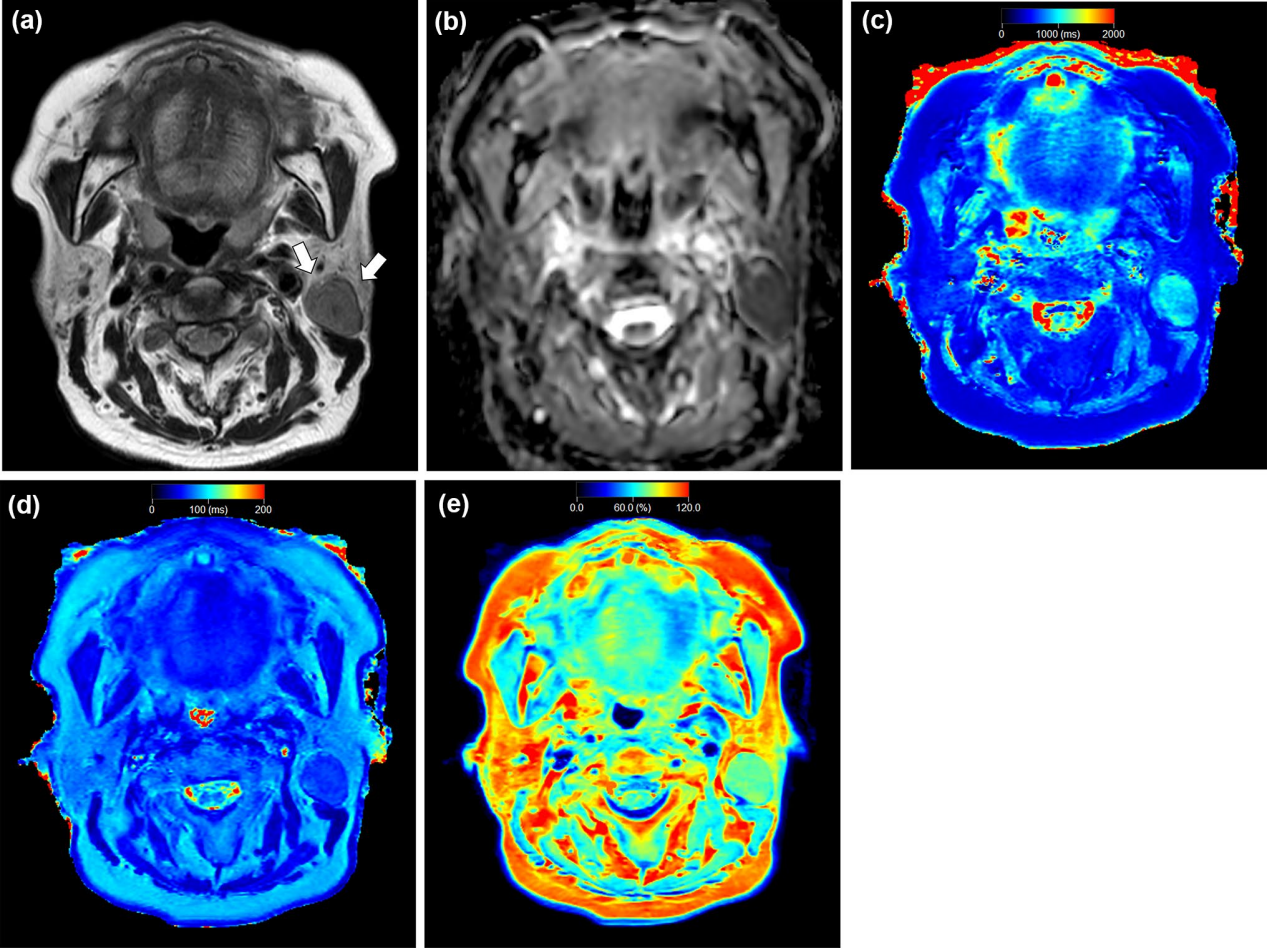
A 65-year-old female with Warthin tumor. T2-weighted image (**a**) shows a lesion with lower signal intensity (arrows) relative to parenchyma in the adjacent left parotid gland. Apparent diffusion coefficient (ADC) (**b**), T1 (**c**), T2 (**d**), and PD (**e**) maps show the lesion, which has a mean ADC value of 0.83 × 10^−3^ mm^2^/s, mean T1 value of 926 ms, mean T2 value of 62 ms, and mean PD value of 73%

**Fig. 5:**
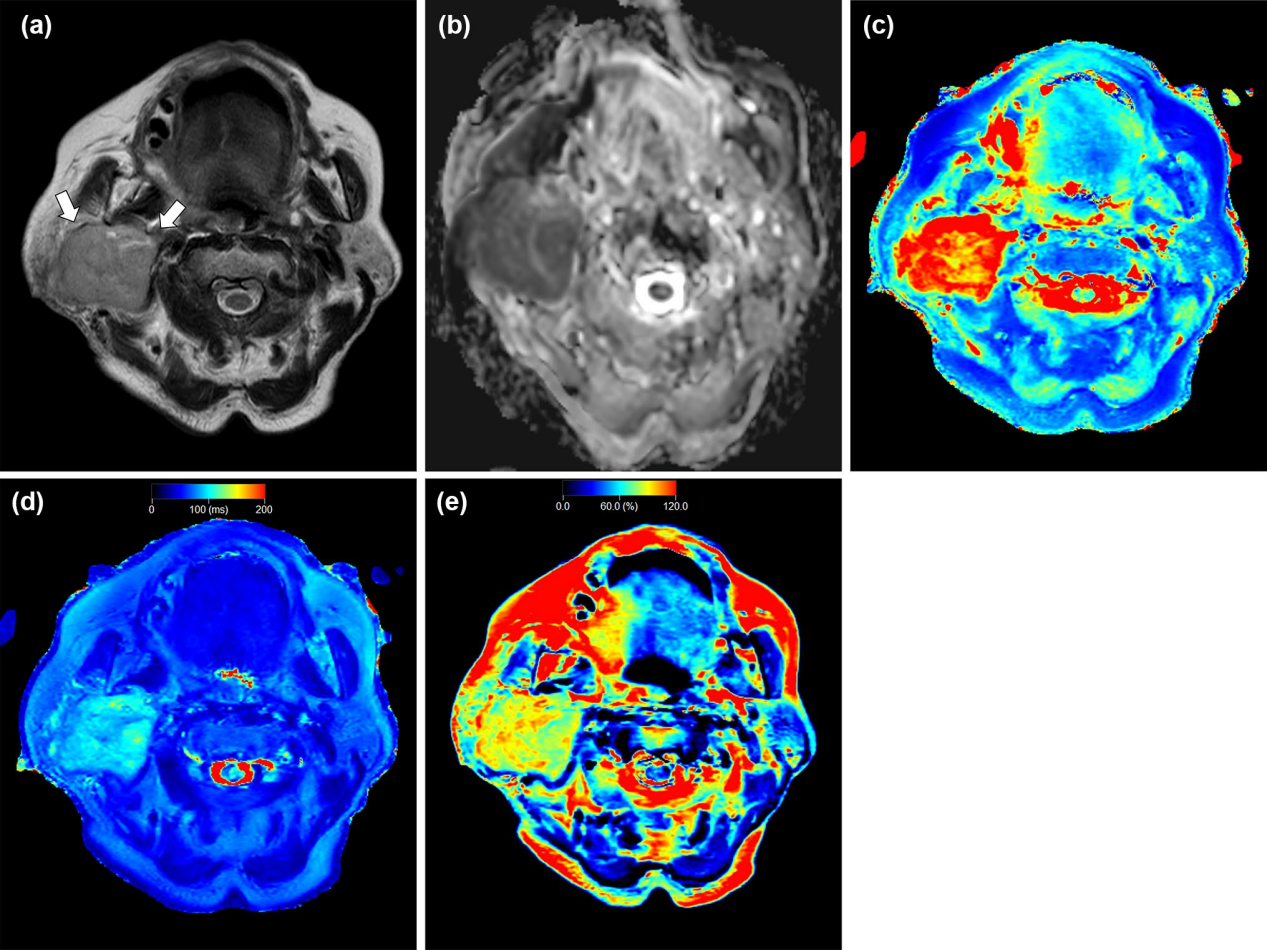
A 72-year-old female with carcinoma ex pleomorphic adenoma. T2-weighted image (**a**) shows a lesion with lower signal intensity (arrows) relative to parenchyma in the adjacent right parotid gland. Apparent diffusion coefficient (ADC) (**b**), T1 (**c**), T2 (**d**), and PD (**e**) maps show the lesion, which has a mean ADC value of 0.77 × 10^−3^ mm^2^/s, mean T1 value of 1869 ms, mean T2 value of 92 ms, and mean PD value of 86%

Representative salivary gland lesions are shown in Figs. 3, 4 and 5.

## Discussion

The major findings of the present study are that PAs demonstrated significantly higher T2 and ADC values compared to MTs and WTs, and that WTs had significantly lower T1 and PD values compared to MTs. Moreover, the combination of T1 with T2 or ADC, and of PD with T2 or ADC, achieved accuracy of 84.4% and 86.7%, respectively, in differentiating MTs from BTs. The present findings suggest that quantitative parameters derived from synthetic MRI can contribute to the accurate diagnosis of salivary gland lesions.

Synthetic MRI is increasingly being utilized in diagnosing various organ conditions. Quantitative parameters such as T1, T2, and PD values derived from synthetic MRI have been found to be effective in differentiation between benign and malignant lesions [17, 18, 20, 21], histological grading [22], and prognosis prediction [23]. In addition to their known efficacy in evaluating myocardial tissues, the application of T1 values has recently been expanded beyond cardiovascular imaging [24–27]. It has been reported that the T1 values of malignant lesions are lower than those of benign lesions and normal tissues in nasopharyngeal [24], thyroid [25], liver [26], and prostate lesions [18, 27]. This difference could be attributed to the increased parenchymal tissue, narrowed extracellular space, and reduced free water content typically found in malignant tumors. The present results demonstrated that MTs had significantly lower T1 values than those of PAs, and that WTs had lower T1 values than those of PAs and MTs, consistent with the findings of a recent report [28]. Another recent report [4] has demonstrated that signal intensity of WTs on T1-weighted images was significantly higher than those of PAs and MTs, which also supports our results. Despite the benign nature of WTs, these lesions exhibited the lowest T1 values, which is probably associated with their abundant lymphocytes and lymphoid interstitium. The microcystic components in WTs that contain accumulations of proteinaceous fluid along with foamy cells, red blood cells, and neutrophils [29–31] could further contribute to the decreased T1 values. Significant differences in T1 values were observed between the subtypes of salivary gland lesions, suggesting the potential of this parameter as a quantitative imaging biomarker of the physical properties of tissue in diagnosing salivary gland lesions.

T2 mapping, which was applied initially for studies of osteoarthritis [32] and myocardial edema [33], has recently been adopted for tumor studies. This technique is especially effective in improving characterization of liver [34] and breast tumors [35, 36]. In the present study, PAs exhibited significantly higher T2 values compared to both MTs and WTs, whereas there was no significant difference in T2 values between MTs and WTs. These findings are consistent with previous reports [37, 38]. PAs exhibited the highest T2 values, which could be related to their abundant stroma such as mucinous-like, myxoid, and chondroid matrices with a high content of free water molecules. The wide range of T2 values observed in PAs could also be linked to the variable proportions of mucinous-like tissue within these tumors.

PD imaging derived from synthetic MRI has shown significant potential in the qualitative diagnosis of lesions. It has been reported that PD values derived from synthetic MRI are lower in malignant lesions than benign lesions in the breast [21] and in lymph nodes [39]. The poorer the tumor differentiation, the lower the PD value in rectal cancer [23] and head and neck squamous cell carcinomas [40, 41]. In our study, PD values were significantly lower in WTs than in both PAs and MTs. The PD values mainly correlate with the concentration of water protons (mobile hydrogen atoms) within each voxel of tissue [42, 43]. The high cellular density within WT lesions, primarily due to abundant lymphocytes, lead to a narrowed extracellular space and reduced free water content. Therefore, PD values in WTs were significantly lower compared to other salivary gland lesions, highlighting the distinctive cellular composition of these tumors. PD value may be a reliable predictor to distinguish WTs from other salivary gland lesions. This study is the first investigation of the potential of PD for diagnosing salivary gland lesions, paving the way for future research in this area.

By integrating various imaging parameters derived from synthetic MRI, our approach provides a comprehensive assessment of the microenvironment of the tumor. This multi-faceted analysis offers valuable insights into the tumor’s cellular structure and tissue composition, enabling more accurate differentiation between benign and malignant lesions [21, 39]. In our study, the integration of parameters derived from synthetic MRI and ADC achieved high accuracy in differentiating MTs from BTs, due to the efficient capture of histological characteristics of PAs and WTs through the combined use of T1, T2, and PD values, and was effective in the differentiation of salivary gland tumors. The increased diagnostic accuracy afforded by multiparametric methods can significantly improve treatment planning and provide the basis for more tailored and more effective therapeutic strategies. This approach represents a substantial advancement in the field of salivary gland tumor diagnosis, potentially resulting in better outcomes for patients through more informed clinical decision-making. Furthermore, the methodology employed in this study acquires multiple parameters in a short time and eliminates the need for contrast agents, and is therefore both time- and cost-effective.

The major limitations of this study are its retrospective study design and the relatively small number of malignant lesions. In addition, the salivary gland malignancies include malignant lymphomas and sarcomas while benign tumors include schwannomas. These differ from the tissue types originating from salivary gland tissue, which may result in heterogeneous data. To validate our results, it will be essential to conduct a prospective study structured to include a more extensive case collection encompassing a broader spectrum of tumor types of salivary gland origin. Moreover, as this research was conducted at a single center, it is crucial to contrast and corroborate our findings with those from various institutions, ideally in a large multicenter study. Variability in equipment, vendors, and MRI settings can notably influence the quantitative measures evaluated in this study. Another limitation of our study is the absence of direct comparisons between parameters obtained from synthetic MRI and findings from conventional MRI studies. Future investigations should involve a larger number of cases to compare the diagnostic capabilities of the methods reported previously with those identified in this study. Additionally, among the cases included in our study, 17 had undergone a biopsy prior to MRI imaging. This could be a potential limitation of our study. However, the ROIs for our analysis were carefully selected to include areas with minimal degenerative changes. Therefore, we believe that the impact of prior biopsies on our findings is limited. Finally, measuring all parameters within a ROI (avoiding obvious cystic or necrotic areas) might have introduced selection bias and affected the consistent acquisition of reliable values for each parameter.

In conclusion, quantitative parameters derived from synthetic MRI were significantly different among MTs, PAs, and WTs. These parameters and their combination can help diagnose such lesions, including differentiation of malignant from benign lesions. These findings offer valuable insights for accurate tumor characterization and are clinically relevant in terms of aiding in early diagnosis and developing tailored treatment strategies.

### Supplementary Information

Below is the link to the electronic supplementary material.

Supplementary file1 (TIF 2025 KB)

## Abbreviations

PA: Pleomorphic adenoma
WT: Warthin tumor
MT: Malignant tumor
BT: Benign tumor
PD: Proton density
ADC: Apparent diffusion coefficient
DWI: Diffusion-weighted imaging
MRI: Magnetic resonance imaging
QRAPMASTER: Quantification of relaxation times and proton density by multi-echo acquisition of saturation recovery using turbo spin-echo readout
TR: Repetition time
TE: Echo time
FOV: Field of view
ROI: Region of interest
ICC: Intraclass correlation coefficient
ROC: Receiver-operating characteristic
AUC: Area under the ROC curve

## References

[1] Sood S, McGurk M, Vaz F. Management of salivary gland tumours: United Kingdom National Multidisciplinary Guidelines. J Laryngol Otol. 2016;130:S142–9. 27841127 10.1017/S0022215116000566 PMC4873929

[2] Bonavolontà P, Germano C, Committeri U, Orabona GD, Piombino P, Abbate V, et al. Surgical management of Warthin tumor: long-term follow-up of 224 patients from 2002 to 2018. Oral Maxillofac Surg. 2024;28:131–6. 37191772 10.1007/s10006-023-01156-4 PMC10914882

[3] Vergez S, Fakhry N, Cartier C, Kennel T, Courtade-Saidi M, Uro-Coste E, et al. Guidelines of the French Society of Otorhinolaryngology-Head and Neck Surgery (SFORL), part I: Primary treatment of pleomorphic adenoma. Eur Ann Otorhinolaryngol Head Neck Dis. 2021;138:269–74. 33060032 10.1016/j.anorl.2020.09.002

[4] Wei PY, Shao C, Huan T, Wang HB, Ding ZX, Han ZJ. Diagnostic value of maximum signal intensity on T1-weighted MRI images for differentiating parotid gland tumours along with pathological correlation. Clin Radiol. 2021;76:472.e19-72.e25. 33731262 10.1016/j.crad.2021.02.011

[5] Geiger JL, Ismaila N, Beadle B, Caudell JJ, Chau N, Deschler D, et al. Management of salivary gland malignancy: ASCO guideline. J Clin Oncol. 2021;39:1909–41. 33900808 10.1200/JCO.21.00449

[6] Di Santo D, Bramati C, Festa BM, Pace GM, Comini LV, Luparello P, et al. Current evidence on diagnosis and treatment of parotid gland lymphomas: a systematic review. Eur Arch Otorhinolaryngol. 2023;280:5219–27. 37638999 10.1007/s00405-023-08206-3

[7] Freling NJ, Molenaar WM, Vermey A, Mooyaart EL, Panders AK, Annyas AA, et al. Malignant parotid tumors: clinical use of MR imaging and histologic correlation. Radiology. 1992;185:691–6. 1438746 10.1148/radiology.185.3.1438746

[8] Christe A, Waldherr C, Hallett R, Zbaeren P, Thoeny H. MR imaging of parotid tumors: typical lesion characteristics in MR imaging improve discrimination between benign and malignant disease. AJNR Am J Neuroradiol. 2011;32:1202–7. 21724574 10.3174/ajnr.A2520 PMC7966029

[9] Kim SY, Borner U, Lee JH, Wagner F, Tshering Vogel DW. Magnetic resonance imaging of parotid gland tumors: a pictorial essay. BMC Med Imaging. 2022;22:191. 36344914 10.1186/s12880-022-00924-0 PMC9641923

[10] Kato H, Kawaguchi M, Ando T, Mizuta K, Aoki M, Matsuo M. Pleomorphic adenoma of salivary glands: common and uncommon CT and MR imaging features. Jpn J Radiol. 2018;36:463–71. 29845358 10.1007/s11604-018-0747-y

[11] Okahara M, Kiyosue H, Hori Y, Matsumoto A, Mori H, Yokoyama S. Parotid tumors: MR imaging with pathological correlation. Eur Radiol. 2003;13(Suppl 4):L25-33. 15018162 10.1007/s00330-003-1999-0

[12] Yabuuchi H, Matsuo Y, Kamitani T, Setoguchi T, Okafuji T, Soeda H, et al. Parotid gland tumors: can addition of diffusion-weighted MR imaging to dynamic contrast-enhanced MR imaging improve diagnostic accuracy in characterization? Radiology. 2008;249:909–16. 18941162 10.1148/radiol.2493072045

[13] Yabuuchi H, Fukuya T, Tajima T, Hachitanda Y, Tomita K, Koga M. Salivary gland tumors: diagnostic value of gadolinium-enhanced dynamic MR imaging with histopathologic correlation. Radiology. 2003;226:345–54. 12563124 10.1148/radiol.2262011486

[14] Takumi K, Fukukura Y, Hakamada H, Ideue J, Kumagae Y, Yoshiura T. Value of diffusion tensor imaging in differentiating malignant from benign parotid gland tumors. Eur J Radiol. 2017;95:249–56. 28987676 10.1016/j.ejrad.2017.08.013

[15] Takumi K, Nagano H, Kikuno H, Kumagae Y, Fukukura Y, Yoshiura T. Differentiating malignant from benign salivary gland lesions: a multiparametric non-contrast MR imaging approach. Sci Rep. 2021;11:2780. 33531644 10.1038/s41598-021-82455-2 PMC7854671

[16] Hagiwara A, Warntjes M, Hori M, Andica C, Nakazawa M, Kumamaru KK, et al. SyMRI of the brain: rapid quantification of relaxation rates and proton density, with synthetic MRI, automatic brain segmentation, and myelin measurement. Invest Radiol. 2017;52:647–57. 28257339 10.1097/RLI.0000000000000365 PMC5596834

[17] Li X, Fan Z, Jiang H, Niu J, Bian W, Wang C, et al. Synthetic MRI in breast cancer: differentiating benign from malignant lesions and predicting immunohistochemical expression status. Sci Rep. 2023;13:17978. 37864025 10.1038/s41598-023-45079-2 PMC10589282

[18] Cui Y, Han S, Liu M, Wu PY, Zhang W, Zhang J, et al. Diagnosis and grading of prostate cancer by relaxation maps from synthetic MRI. J Magn Reson Imaging. 2020;52:552–64. 32027071 10.1002/jmri.27075

[19] Zhu K, Chen Z, Cui L, Zhao J, Liu Y, Cao J. The preoperative diagnostic performance of multi-parametric quantitative assessment in rectal carcinoma: a preliminary study using synthetic magnetic resonance imaging. Front Oncol. 2022;12: 682003. 35707367 10.3389/fonc.2022.682003 PMC9190242

[20] Zhang Z, Li S, Wang W, Zhang Y, Wang K, Cheng J, et al. Synthetic MRI for the quantitative and morphologic assessment of head and neck tumors: a preliminary study. Dentomaxillofac Radiol. 2023;52:20230103. 37427697 10.1259/dmfr.20230103 PMC10461255

[21] Gao W, Zhang S, Guo J, Wei X, Li X, Diao Y, et al. Investigation of synthetic relaxometry and diffusion measures in the differentiation of benign and malignant breast lesions as compared to BI-RADS. J Magn Reson Imaging. 2021;53:1118–27. 33179809 10.1002/jmri.27435

[22] Cai Q, Wen Z, Huang Y, Li M, Ouyang L, Ling J, et al. Investigation of synthetic magnetic resonance imaging applied in the evaluation of the tumor grade of bladder cancer. J Magn Reson Imaging. 2021;54:1989–97. 34080268 10.1002/jmri.27770

[23] Ma L, Lian S, Liu H, Meng T, Zeng W, Zhong R, et al. Diagnostic performance of synthetic magnetic resonance imaging in the prognostic evaluation of rectal cancer. Quant Imaging Med Surg. 2022;12:3580–91. 35782274 10.21037/qims-22-24 PMC9246756

[24] Yang F, Li Y, Li X, Yu X, Zhao Y, Li L, et al. The utility of texture analysis based on quantitative synthetic magnetic resonance imaging in nasopharyngeal carcinoma: a preliminary study. BMC Med Imaging. 2023;23:15. 36698156 10.1186/s12880-023-00968-w PMC9875491

[25] Yuan L, Zhao P, Lin X, Yu T, Diao R, Ning G. T1 mapping and reduced field-of-view DWI at 3.0 T MRI for differentiation of thyroid papillary carcinoma from nodular goiter. Clin Physiol Funct Imaging. 2023;43:137–45. 36440541 10.1111/cpf.12803

[26] Wang F, Yang Q, Zhang Y, Liu J, Liu M, Zhu J. 3D variable flip angle T1 mapping for differentiating benign and malignant liver lesions at 3T: comparison with diffusion weighted imaging. BMC Med Imaging. 2022;22:146. 35982406 10.1186/s12880-022-00873-8 PMC9389795

[27] Baur ADJ, Hansen CM, Rogasch J, Posch H, Elezkurtaj S, Maxeiner A, et al. Evaluation of T1 relaxation time in prostate cancer and benign prostate tissue using a Modified Look-Locker inversion recovery sequence. Sci Rep. 2020;10:3121. 32080281 10.1038/s41598-020-59942-z PMC7033189

[28] Wen B, Zhang Z, Fu K, Zhu J, Liu L, Gao E, et al. Value of pre-/post-contrast-enhanced T1 mapping and readout segmentation of long variable echo-train diffusion-weighted imaging in differentiating parotid gland tumors. Eur J Radiol. 2023;162: 110748. 36905715 10.1016/j.ejrad.2023.110748

[29] Kato H, Kanematsu M, Watanabe H, Mizuta K, Aoki M. Salivary gland tumors of the parotid gland: CT and MR imaging findings with emphasis on intratumoral cystic components. Neuroradiology. 2014;56:789–95. 24948426 10.1007/s00234-014-1386-3

[30] Ikeda M, Motoori K, Hanazawa T, Nagai Y, Yamamoto S, Ueda T, et al. Warthin tumor of the parotid gland: diagnostic value of MR imaging with histopathologic correlation. AJNR Am J Neuroradiol. 2004;25:1256–62. 15313720 PMC7976549

[31] Minami M, Tanioka H, Oyama K, Itai Y, Eguchi M, Yoshikawa K, et al. Warthin tumor of the parotid gland: MR-pathologic correlation. AJNR Am J Neuroradiol. 1993;14:209–14. 8427092 PMC8334432

[32] Hada S, Ishijima M, Kaneko H, Kinoshita M, Liu L, Sadatsuki R, et al. Association of medial meniscal extrusion with medial tibial osteophyte distance detected by T2 mapping MRI in patients with early-stage knee osteoarthritis. Arthritis Res Ther. 2017;19:201. 28899407 10.1186/s13075-017-1411-0 PMC5596458

[33] Tahir E, Sinn M, Bohnen S, Avanesov M, Säring D, Stehning C, et al. Acute versus chronic myocardial infarction: diagnostic accuracy of quantitative native T1 and T2 mapping versus assessment of edema on standard T2-weighted cardiovascular MR images for differentiation. Radiology. 2017;285:83–91. 28678672 10.1148/radiol.2017162338

[34] Cieszanowski A, Anysz-Grodzicka A, Szeszkowski W, Kaczynski B, Maj E, Gornicka B, et al. Characterization of focal liver lesions using quantitative techniques: comparison of apparent diffusion coefficient values and T2 relaxation times. Eur Radiol. 2012;22:2514–24. 22699872 10.1007/s00330-012-2519-x PMC3472073

[35] Liu L, Yin B, Shek K, Geng D, Lu Y, Wen J, et al. Role of quantitative analysis of T2 relaxation time in differentiating benign from malignant breast lesions. J Int Med Res. 2018;46:1928–35. 29557239 10.1177/0300060517721071 PMC5991255

[36] Micek M, Aebisher D, Surówka J, Bartusik-Aebisher D, Madera M. Applications of T(1) and T(2) relaxation time calculation in tissue differentiation and cancer diagnostics-a systematic literature review. Front Oncol. 2022;12:1010643. 36531030 10.3389/fonc.2022.1010643 PMC9749890

[37] Wu Q, Zhu LN, Jiang JS, Bu SS, Xu XQ, Wu FY. Characterization of parotid gland tumors using T2 mapping imaging: initial findings. Acta Radiol. 2020;61:629–35. 31542938 10.1177/0284185119875646

[38] Baohong W, Jing Z, Zanxia Z, Kun F, Liang L, Eryuan G, et al. T2 mapping and readout segmentation of long variable echo-train diffusion-weighted imaging for the differentiation of parotid gland tumors. Eur J Radiol. 2022;151: 110265. 35472650 10.1016/j.ejrad.2022.110265

[39] Wang P, Hu S, Wang X, Ge Y, Zhao J, Qiao H, et al. Synthetic MRI in differentiating benign from metastatic retropharyngeal lymph node: combination with diffusion-weighted imaging. Eur Radiol. 2023;33:152–61. 35951044 10.1007/s00330-022-09027-4

[40] Yang F, Li Y, Lei H, Wei H, Du Q, Yu X, et al. Histogram analysis of synthetic magnetic resonance imaging: Correlations with histopathological factors in head and neck squamous cell carcinoma. Eur J Radiol. 2023;160: 110715. 36753947 10.1016/j.ejrad.2023.110715

[41] Yang F, Li X, Li Y, Lei H, Du Q, Yu X, et al. Histogram analysis of quantitative parameters from synthetic MRI: correlations with prognostic factors in nasopharyngeal carcinoma. Eur Radiol. 2023;33:5344–54. 37036478 10.1007/s00330-023-09553-9

[42] Mezer A, Rokem A, Berman S, Hastie T, Wandell BA. Evaluating quantitative proton-density-mapping methods. Hum Brain Mapp. 2016;37:3623–35. 27273015 10.1002/hbm.23264 PMC6204063

[43] Gracien RM, Reitz SC, Hof SM, Fleischer V, Zimmermann H, Droby A, et al. Changes and variability of proton density and T1 relaxation times in early multiple sclerosis: MRI markers of neuronal damage in the cerebral cortex. Eur Radiol. 2016;26:2578–86. 26494641 10.1007/s00330-015-4072-x

