# Supplementary figures and images for: Multiparametric approach with synthetic MR imaging for diagnosing salivary gland lesions
Source: Jpn J Radiol. 2024 May 11;42(9):983–92. doi: 10.1007/s11604-024-01578-4 (PMC11364709; doi:10.1007/s11604-024-01578-4)

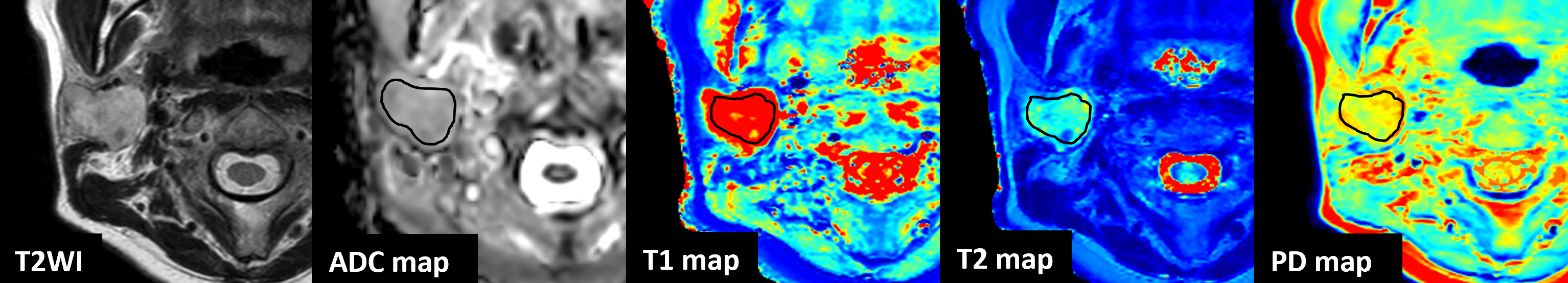

Supplement: Supplementary file 1 — Supplementary file1 (TIF 2025 KB) [file 11604_2024_1578_MOESM1_ESM.tif]
